# Prognostic value of the autophagy markers LC3 and p62/SQSTM1 in early-stage non-small cell lung cancer

**DOI:** 10.18632/oncotarget.9647

**Published:** 2016-05-26

**Authors:** Anna M. Schläfli, Olivia Adams, José A. Galván, Mathias Gugger, Spasenija Savic, Lukas Bubendorf, Ralph A. Schmid, Karl-Friedrich Becker, Mario P. Tschan, Rupert Langer, Sabina Berezowska

**Affiliations:** ^1^ Institute of Pathology, University of Bern, Bern, Switzerland; ^2^ Graduate School for Cellular and Biomedical Sciences, University of Bern, Bern, Switzerland; ^3^ Promed Laboratoire Médical, Marly, Switzerland; ^4^ Institute of Pathology, Universty Hospital Basel, Basel, Switzerland; ^5^ Division of General Thoracic Surgery, Inselspital University Hospital Bern, Bern, Switzerland; ^6^ Institute of Pathology, Technische Universität München, München, Germany

**Keywords:** autophagy, LC3, p62/SQSTM1, immunohistochemistry, non-small cell lung cancer

## Abstract

Autophagy is a cellular degrading process that promotes tumor cell survival or cell death in cancer, depending on the progress of oncogenesis. Protein light chain 3 (LC3) and p62/SQSTM1 (p62) are associated with autophagosomal membranes that engulf cytoplasmic content for subsequent degradation. We studied LC3 and p62 expression using immunohistochemistry in a large cohort of 466 stage I/II non-small cell lung cancer (NSCLC) using a tissue microarray. We evaluated dot-like cytoplasmic expression of LC3 and dot-like, cytoplasmic and nuclear staining for p62 in relation to clinico-pathological parameters.

LC3 expression correlated with all p62 patterns, as those correlated among each other (*p* < 0.001 each). There was no correlation with stage, age or gender. A combination of high LC3/high p62 dot-like staining (suggesting impaired autophagy) showed a trend for better outcome (*p* = 0.11). Interestingly, a combined low cytoplasmic/low nuclear p62 expression regardless of dot-like staining was an independent prognostic factor for longer survival (*p* = 0.006; HR=1.96), in addition to tumor stage (*p* = 0.004; HR=1.4).

The autophagy markers LC3 and p62 are differentially expressed in NSCLC, pointing towards a biologically significant role. High LC3 levels seem to be linked to lower tumor aggressiveness, while high general p62 expression was significantly associated with aggressive tumor behavior.

## INTRODUCTION

Lung cancer is one of the most frequent cancers, and responsible for the most deaths due to malignant disease. The desire for better understanding its biology, which could ideally confer additional, better and more precise oncological therapeutics, is thus enormous. Non-small cell lung cancer (NSCLC) comprises the majority of lung carcinomas. Most of NSCLC can be histologically typed as adenocarcinoma (AC), squamous cell carcinoma (SqCC) or large cell carcinoma (LCC), as defined by the WHO [1].

The cellular process of autophagy has many functions including bulk degradation of cytoplasmic content to ensure homeostasis [2], degradation of superfluous or damaged organelles [3] and the engulfment of pathogens [4]. The role of autophagy in cancer is complex and depends on tumor stage, type and the driving oncogene [5–7]. Under homeostatic conditions autophagy is regarded as a tumor suppressor process, but there is evidence it is later on required for tumor progression [8, 9].

Exploiting autophagy for anti-cancer therapeutic benefit is an area gaining ever increasing focus. On a cellular level autophagy aims at degrading cytoplasmic content in the lysosomal compartment [2]. Macroautophagy, referred to as autophagy from here on, is hallmarked by the formation of double-membrane vesicles, so-called autophagosomes, which finally fuse with lysosomes to degrade their content [10].

On a molecular level several functional complexes, comprising so-called autophagy related (*ATG*) genes, are involved in autophagosome biogenesis, cargo recognition and in mediating autophagosome-lysosome fusion. Briefly, the unc-51-like kinase 1 (ULK1) complex is important for autophagy initiation. Down-stream of the ULK-1 complex the coiled-coil myosin-like BCL2-interacting protein (Beclin1) complex is generating phosphoinositol-3-phosphates (PI3Ps), allowing for the recruitment of other ATG proteins [11]. Furthermore, two ubiquitin-like systems are required for vesicle elongation and cargo recognition, namely the ATG12-ATG5- and the ATG8-conjugation system. The latter one serves to lipidate ATG8 family members, including microtubule-associated protein 1 light chain 3 (MAP1LC3 or LC3, hereafter referred to as LC3) [12, 13]. LC3 comprises three isoforms LC3A, B and C. Since LC3 is incorporated into the inner and outer membrane of autophagosomes [14], its detection allows for monitoring the autophagy pathway [15]. LC3-I is cytosolic, whereas the lipidated form LC3-II is membrane bound [14].

Autophagy can either be non-selective or can very specifically target certain portions of the cytoplasm for degradation [16]. Different autophagy receptors are mediating selectivity by being able to interact with the cargo on the one hand and the autophagy machinery on the other hand [17]. The best studied is sequestosome 1 (SQSTM1 or p62, hereafter referred to as p62). Essential for its function as an autophagy receptor is its ability to interact with ubiquitinated cargo and LC3B [18]. During this process p62 itself is constantly degraded. Reduced levels of p62 are therefore associated with an activated autophagy pathway [15].

Both LC3 and p62 are frequently used as markers to assess autophagy [15]. Although autophagy is a flux, and should ideally be measured in functional assays, immunohistochemistry is the method of choice for tissue based retrospective analysis of large cohorts. Thereby, dot-like staining of LC3 serves as a surrogate marker for autophagic vesicles. As p62 is constantly degraded in autolysosomes, it is a surrogate marker for autophagic degradation.

In NSCLC, there are only very few studies on the prognostic significance of the expression of autophagy markers, and all have been conducted in broad stage collectives [19, 20]. Because the role of autophagy in cancer may be stage dependent, the aim of our present study was to assess autophagy-associated markers in non-metastasized, early-stage NSCLC.

## RESULTS

### Staining patterns

Formalin-fixed and paraffin embedded (FFPE) tissue of 466 primary resected, chemotherapy-naïve, early-stage NSCLC was analyzed for the expression of autophagy associated markers LC3 and p62. The anti-LC3 antibody from Novus (N) is known to recognize the LC3 isoforms A and B [21] the anti-LC3B antibody from Cell Signaling (CS) is isoform B specific [22].

Staining of both antibodies could be assessed in 442 cases, and correlated significantly (*p* < 0.0001), although the CS antibody showed a generally weaker staining, with few positive cases. LC3 (CS) dot-like staining could be evaluated in 464 cases: score 0 in 403 cases (86.9%), score 1 in 47 cases (10.1%), score 2 in 11 cases (2.4%), score 3 in 3 cases (0.6%).

**Figure 1:**
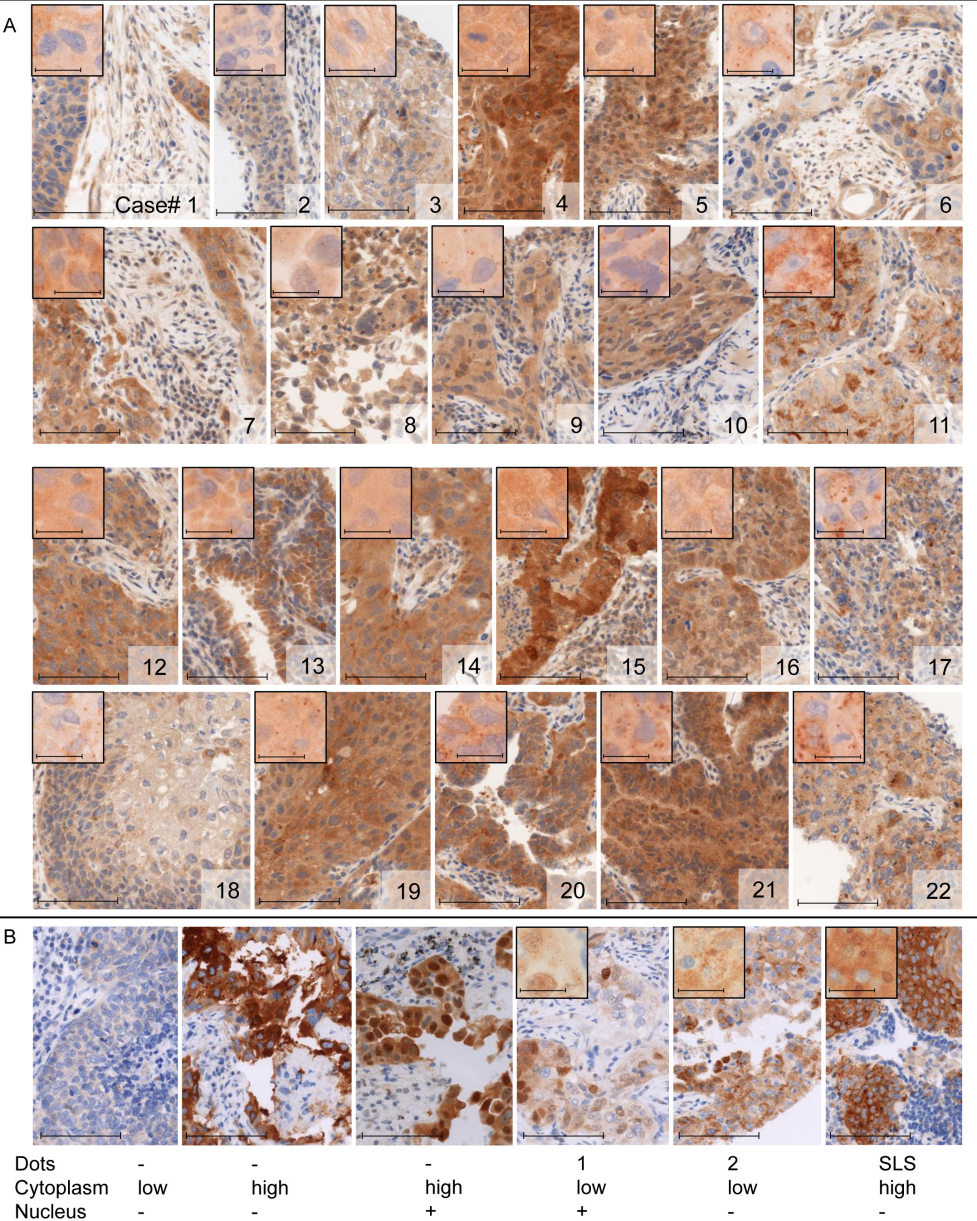
Immunohistochemical staining of LC3 and p62 **A.** LC3 (Novus) staining patterns of the 22 cases further analysed by immunoblot analysis (figure 2). Results of dot-like staining patterns are given in the upper panels of figure 2A and B for each case. **B.** Examples of p62 staining, with cytoplasmic, dot-like, nuclear and SLS staining patterns. (original magnification 400 x, scale bar 100 μm; insets: original magnification 1000 x, scale bar 20 μm).

LC3 (N) dot-like staining could be evaluated in 443 cases and was observed as score 0 in 240 cases (54.2%), score 1 in 120 cases (27.1%), score 2 in 48 cases (10.8%) and score 3 in 35 cases (7.9%)(Figure 1A). Stone-like structures (SLS)[20] were present in 8 cases (1.8%). For p62 dot-like staining, 420 punches were suitable for evaluation, and 328 cases (78.1%) scored 0, 59 cases (14%) scored 1, 24 cases (5.7%) scored 2 and 9 cases (2.1%) scored 3. SLS were present in 14 cases (3.3%). Cytoplasmic staining of p62 was absent (score 0) in 82 cases (19.5%), score 1 in 266 cases (63.3%) and score 2 in 72 cases (17.1%). Nuclear staining was absent in 264 cases (62.9%) and present in 156 cases (37.1%).

LC3 dot-like staining showed a significant correlation with all p62 staining patterns (*p* < 0.001 each), and all p62 staining patterns correlated among each other (*p* < 0.001 each). Examples for p62 cytoplasmic, dot-like, nuclear and SLS staining are shown in Figure 1B.

Assessment of staining heterogeneity was performed in 38 exemplary cases, using 8 punches from two tissue blocks of each tumor. Completely homogenous staining with regard to score 1 to 3 was observed in 12/38 cases (31.6%) for LC3 dot-like staining, 13/38 cases (34.2%) for p62 dot-like staining, 18/38 cases (47.4%) for p62 cytoplasmic staining and 19/38 cases (50%) for p62 nuclear staining. Heterogeneous staining, however, with a deviation of > 1 scoring points in > 1 spot, that would also have caused a different classification into „low“ and „high“ as described below (refer to following paragraph *Correlation with clinico-pathologic parameters*) was observed in only 2/38 cases (5.3%) for LC3 dot-like staining, 1/38 cases (2.6%) for p62 dot-like staining, no case for p62 cytoplasmic staining and 1/38 cases (2.6%) for p62 nuclear staining.

### Immunoblot analysis

**Figure 2:**
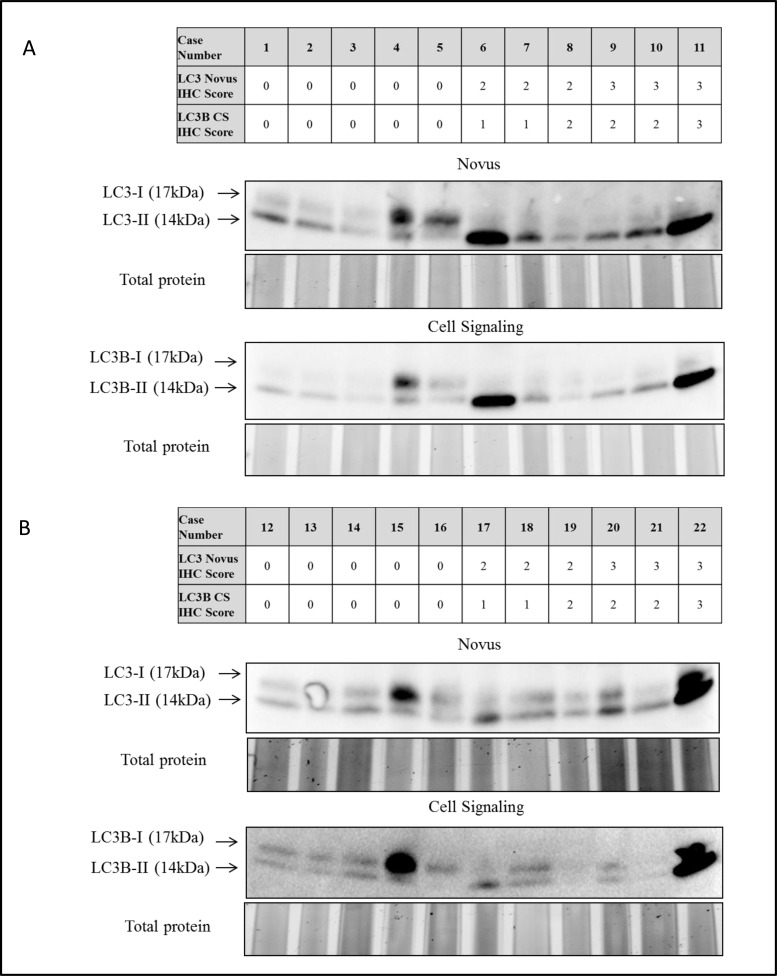
Immunoblot analysis of LC3 in protein extracts from FFPE tissue from 22 early-stage non-small cell lung carcinomas **A.** Immunoblot analysis of LC3-I (cytosolic) and LC3-II (membrane-bound) of cases 1 to 12, and **B.** cases 13 to 22, using anti-LC3 Novus (middle panel) and anti-LC3B Cell Signaling (bottom panel) primary antibodies. Total protein was visualised and used as loading control. Immunohistochemistry scores for anti-LC3 Novus and anti-LC3B Cell Signaling primary antibodies are shown in the tables (upper panel).

During the process of autophagosome formation LC3 gets cleaved and lipidated before incorporation into the membrane. This modification enables to distinguish between cytosolic LC3 (LC3-I) and membrane-bound LC3 (LC3-II) on a western blot [23]. Immunoblot analysis of 22 cases selected according to absent and strongly present LC3 dot-like staining revealed the feasibility of this methodology for LC3 evaluation in FFPE tissue. The results correlated mostly with the immunohistochemical staining patterns as depicted in Figure 1 *versus* 2. Importantly, both LC3 antibodies (from Novus and Cell Signaling) showed equal results on Western Blot (Figure 2).

### Correlation with clinico-pathologic parameters

For the purposes of correlation with pathological and clinical parameters, immunohistochemistry scores were categorized as either “low” or “high” for each staining pattern according to our established protocol [24] with slight modifications, following the prognostic impact of the single scores: For LC3 dot-like staining scores 0 and 1 were classified as low, scores 2 and 3 as high. For p62 dot-like staining score 0 was classified as low, scores 1, 2 and 3 were interpreted as high. For p62 cytoplasmic staining scores 0 and 1 were classified as low and score 2 as high. Only 14 cases (3%) showed high dot-like staining using LC3 (CS), 83 cases (18.7%) using LC3 (N).

Low LC3 (N) dot-like staining was more frequent in males (*p* = 0.016) and SqCC (*p* = 0.017). There was no association with age (median), pT category or stage (Table 1). There was no significant association with the abovementioned factors for p62 dot-like staining or the presence of LC3 positive or p62 positive SLS. In contrast, lower p62 cytoplasmic and nuclear stainings were more frequent in AC (*p* = 0.029 and *p* < 0.001, respectively; Table 2).

**Table 1:** LC3 (Novus) and p62 dot-like staining and clinico-pathologic parameters (total *n* = 466; for evaluation LC3 *n* = 443; p62 *n* = 420)

parameter		total	LC3 dot-like staining			p62 dot-like staining		
		N (%)	low	high	*p*	low	high	*p*
**total**		466 (100)	360	83		328	92	
**age**								
	***<67 years***	247 (53)	191	47	0.556	169	53	0.302
	***≥ 67 years***	219 (47)	169	36		159	39	
**gender**								
	***male***	343 (73.6)	276	53	0.016	237	68	0.753
	***female***	123 (26.4)	84	30		91	24	
**histology**								
	***AdCA***	202 (43.3)	146	48	0.017	144	38	0.463
	***SqCC***	229 (49.1)	186	30		164	45	
	***LCC***	35 (7.5)	28	5		20	9	
**pT category**								
	***1a***	24 (5.2)	17	4	0.789	14	6	0.108
	***1b***	31 (6.7)	23	4		13	9	
	***2a***	304 (65.2)	233	59		217	61	
	***2b***	68 (14.6)	56	9		52	9	
	***3***	39 (8.4)	31	7		32	7	
**UICC stage**								
	***IA***	55 (11.8)	40	8	0.666	27	15	0.076
	***IB***	304 (65.2)	233	59		217	61	
	***IIA***	68 (14.6)	56	9		52	9	
	***IIB***	39 (8.4)	31	7		32	7	

**Table 2:** p62 cytoplasmic and nuclear staining and clinico-pathologic parameters (total *n*=466; for evaluation *n*=420)

parameter		total	p62 cytoplasmic staining			p62 nuclear staining		
		N (%)	low	high	*p*	absent	present	*p*
**total**		466 (100)	348	72		264	156	
**age**								
	***<67 years***	247 (53)	187	35	0.428	143	79	0.442
	***≥ 67 years***	219 (47)	161	37		121	77	
**gender**								
	***male***	343 (73.6)	247	58	0.097	185	120	0.140
	***female***	123 (26.4)	101	14		79	36	
**histology**								
	***AdCA***	202 (43.3)	161	21	0.029	134	48	<0.001
	***SqCC***	229 (49.1)	164	45		112	97	
	***LCC***	35 (7.5)	23	6		18	11	
**pT category**								
	***1a***	24 (5.2)	17	3	0.113	13	7	0.942
	***1b***	31 (6.7)	15	7		15	7	
	***2a***	304 (65.2)	233	45		173	105	
	***2b***	68 (14.6)	47	14		38	23	
	***3***	39 (8.4)	36	3		25	14	
**UICC stage**								
	***IA***	55 (11.8)	32	10	0.145	28	14	0.894
	***IB***	304 (65.2)	233	45		173	105	
	***IIA***	68 (14.6)	47	14		38	23	
	***IIB***	39 (8.4)	36	3		25	14	

### Survival analysis

Survival data was available for 349 patients. Survival analysis showed a better overall survival (OS) and recurrence free survival (RFS) for younger patients (cut-off median; OS *p* = 0.002; RFS *p* = 0.012), for females (OS *p* = 0.06; RFS *p* = 0.035), for patients with AC and LCC (OS *p* = 0.005; RFS *p* = 0.025), and with lower pT categories/UICC stages (OS *p* = 0.004/*p* = 0.003; RFS *p* = 0.018/*p* = 0.137; respectively).

None of the patients with high LC3 (CS) dot-like staining relapsed or died, but short follow up times in this sub-group preclude any conclusions and further analyses.

**Figure 3:**
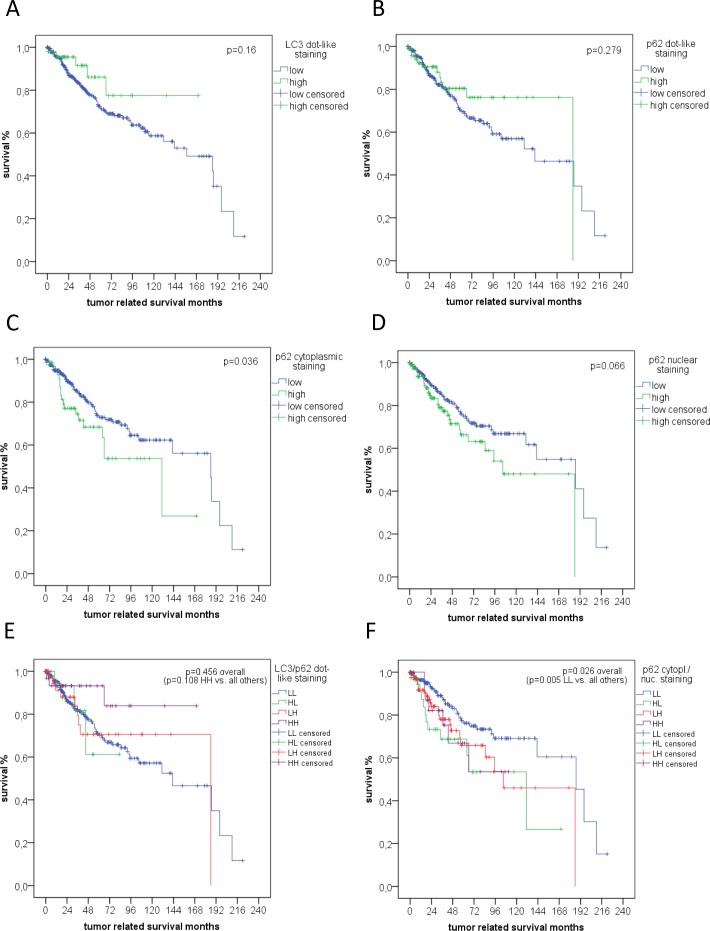
Survival analysis Kaplan Meier curves for tumour related overall survival assessed for **A.** LC3 and **B.** p62 dot-like staining, **C.** cytoplasmic and **D.** nuclear p62 staining, **E.** a combination of LC3/p62 dot-like staining (HH = both high, LL = both low, HL = high LC3/low p62, LH = low LC3/high p62), and **F.** a combination of p62 cytoplasmic and nuclear staining (HH = both high, LL = both low, HL = high cytoplasmic, low nuclear, LH = low cytoplasmic, high nuclear).

For LC3 (N), high dot-like staining patterns were in trend linked to a better OS (*p* = 0.16), similar to high p62 dot-like staining (*p* = 0.28), but not to RFS (*p* = 0.49; *p* = 0.855). The presence of SLS was not associated with survival. In contrast, low p62 cytoplasmic staining was significantly associated with a better tumor related OS (*p* = 0.036), similar to negative p62 nuclear staining (trend; *p* = 0.066)(Figure 3), but not with RFS (*p* = 0.091; *p* = 0.536, respectively).

According to previously published work [25] a combination of LC3 and p62 dot-like staining patterns was analyzed: A small subgroup of tumors with both high LC3 and p62 dot-like staining (*n* = 31) was associated with a better OS. In contrast, high LC3/low p62 dot-like pattern (*n* = 18) and low LC3/any level of p62 dot-like pattern (*n* = 300) had a similarly unfavorable prognostic impact (*p* = 0.11) (Figure 3). This trend was not demonstrated for RFS (*p* = 0.514).

For p62 cytoplasmic and nuclear staining, subgroups were built according to a different recently published paper [26]. Tumors with both p62 low cytoplasmic and nuclear staining (*n* = 185) were associated with a significantly better OS and RFS than mixed (*n* = 139) and both high cytoplasmic and nuclear stained tumors (*n* = 25) (OS *p* = 0.005; RFS *p* = 0.008)

In multivariate analysis including UICC pT category/UICC stage, histological subtype, age, gender and p62 cytoplasmic/nuclear staining, only pT category/UICC stage and low p62 cytoplasmic/nuclear staining were independent prognostic factors for OS (HR = 1.96; 95%CI 1.2-3.2; *p* = 0.006)(Table 3) and RFS (HR = 1.655; 95% CI; 1.1-2.4; *p* = 0.011).

**Table 3:** Multivariate analysis for tumor related overall survival

parameter	HR	95% confidential interval		*p*-value
		min	max	
***Age (median)***	1.574	0.982	2.525	0.06
***Gender***	0.72	0.39	1.33	0.294
***Histology***	1.148	0.765	1.723	0.504
***pT category***	1.407	1.117	1.772	0.004
***p62 cytoplasmic/nuclear***	1.962	1.217	3.164	0.006

## DISCUSSION

We investigated a homogeneous, large early-stage NSCLC cohort of 466 patients for the expression of the autophagy markers LC3 and p62, using a previously validated immunohistochemical protocol [24]. The observed differential expression of both markers points towards a biologically significant role in NSCLC, although drawing conclusions from the staining patterns on specific disruptions of the autophagy mechanism in the tumors is not clear-cut.

A trend for better tumor related OS was seen in tumors expressing high dot-like staining of both LC3 and p62, which in a simplified model could imply impairment at late stages of the autophagy cascade. The low number of tumors expressing high LC3 in our cohort may have precluded statistical significance although the results may be valid and important. Corroborating studies are therefore justified. Contrary to the reported adverse prognostic significance of SLS in the SqCC subgroup of NSCLC, using antibodies to the LC3 isoform LC3A [20], detection of SLS was rare and not associated with outcome in our cohort.

p62 serves as a link between LC3B and ubiquitinated substrates to be degraded in autolysosomes, rendering it a surrogate marker for degradation [15]. Thus, high LC3/low p62 dot-like pattern may be indicative of activated and intact autophagy, and low LC3/p62 any dot-like pattern may show low basal autophagy [25]. Those staining patterns had similarly unfavorable prognostic impact in our collective. However, it is questionable if drawing conclusions from steady-state levels of autophagy markers on the functional state of autophagic activity is a valid approach. As very recently updated, the methodology to assess this highly dynamic process on human FFPE tissue still needs significant improvement [15]. LC3 puncta may mean activated autophagy, but also impaired autophagy due to a late stage block. Second, all autophagy markers may be associated with non-autophagic structures.

Interestingly though, it was high p62 cytoplasmic/nuclear expression that emerged as an independent factor for shorter tumor related OS and RFS, regardless of histological subtype. This is in line with Inoue *et al*., who reported high cytoplasmic expression of p62 to be an independent marker for worse prognosis among AC (*n* = 72), in a cohort of 109 NSCLC, without specifying for nuclear positivity [19]. Similar correlations of high cytoplasmic p62 expression and adverse clinical features were also found in other cancer types, such as breast cancer [27, 28], prostate cancer [29] and oral SqCC [25]. It is important to note, that this cytoplasmic and nuclear expression of p62 may not necessarily be linked to autophagy. One alternative candidate effector may be the NRF2-KEAP1-pathway. Several groups could show that high levels of p62 lead to Nrf2 stabilization and subsequent transcription of genes with an antioxidant function [30]. Importantly, persistent activation of Nrf2 *via* p62 stabilization contributes to tumor progression [31]. However, in the study by Inoue *et al.* accumulation of p62 did not necessarily result in the stabilization of NRF2 [19]. Because increased p62 was shown to lead to NFκB-activation [32, 33], another explanation for worse outcome may be the potentiation of NFκB-dependent transcription *via* p62 [19].

Whereas the cytoplasmic function of p62 is well studied its nuclear function remains less clear. It has been shown that p62 is a protein able to rapidly shuttle between nucleus and cytoplasm, although its preferential localization under homeostatic conditions is cytoplasmic. In the nucleus p62 strongly co-localizes with PML-bodies and is probably involved in delivering ubiquitinated proteins for degradation [34]. Furthermore, p62 is involved in recruiting ALFY, a crucial factor for autophagic degradation of protein aggregates, from the nucleus to the cytoplasm [35]. It might well be that p62 is involved in recruiting other proteins to the cytoplasm as well.

It remains to be investigated whether accumulation of p62 in the nucleus is cancer cell-specific, and if so, what functional consequences it may have. Mislocalization of proteins in cancer cells is seen quite often. The best known is probably the predominant nuclear localization of NFκB in many cancer types [36].

In summary, this is the largest study to date reporting the expression of autophagy related markers LC3 and p62 in a well-defined, early-stage NSCLC cohort of 466 cases. We report the correlation of dot-like immunohistochemical staining for LC3 with LC3-II protein expression assessed by immunoblot analysis of the same FFPE archival cases, corroborating the feasibility to assess autophagy structures using immunohistochemistry.

We observed a trend for better outcome in tumors with high dot-like staining of LC3 and p62, being surrogates for autophagic vacuoles and thus the process of autophagy, although low numbers of this subgroup might have precluded statistical significance. Multivariate analysis rendered cytoplasmic/nuclear p62 staining an independent predictor of worse outcome, regardless of LC3 expression.

Our results warrant further investigations concerning the link between expression data and functional autophagy states and a possible non-autophagy related role of p62 in NSCLC.

## MATERIALS AND METHODS

### Patient cohort

The retrospective study included patients with primary resected node-negative early-stage NSCLC (UICC 7^th^ edition 2009 stage IA-IIB)[37], treated with curative surgery and diagnosed at the Institute of Pathology, University of Bern, Switzerland and the Institute of Pathology, University Hospital Basel, Switzerland between January 1988 and August 2008. The detailed staging work-up for this cohort of 544 patients has already been reported [38]. After exclusion of cases with lymph node metastases, neoadjuvant treatment, rare tumor types other than AC, SqCC and LCC, and insufficient tumor tissue for further analysis, 466 patients were finally included. This retrospective study was approved by the local ethics committee.

Median age of the patient cohort was 67 years (range; 39-83 years). The detailed distribution of clinic-pathologic characteristics can be seen in Tables 1 and 2. The median RFS was 110 months (95% CI; 85-134 months). Tumor related OS, which was calculated from the day of surgical resection until last contact or tumor specific death, was 186 months (95% CI; 119-252 months).

### Tissue microarray

A tissue microarray (TMA) was constructed as reported elsewhere [38]. In short, formalin fixed paraffin embedded (FFPE) tissue blocks were retrieved from the archives of the Institutes of Pathology. One punch (diameter 0.6 mm) from the tumor center of the best-preserved block was transferred to the receptor TMA-block.

To assess for staining heterogeneity, 38 patients with positive and negative LC3 staining patterns were selected from the Bern sub-cohort. A TMA was constructed as reported elsewhere [39], using 8 cores (diameter 0.6 mm) from 2 FFPE blocks per tumor. Additional punches (4 × 1 mm per tumor) were taken for protein extraction.

### Immunohistochemistry

Immunohistochemical staining was performed using the automated system BOND RX (Leica Biosystems, Newcastle, UK). TMA sections were cut at 4 μm, deparaffinized and rehydrated in dewax solution (Leica Biosystems). Endogenous peroxidase activity was blocked with H_2_O_2_ solution for 4 minutes.

All samples were incubated with the following primary antibodies for 30 minutes at room temperature (RT), as described before [24]: LC3B from Cell Signaling Technology (MA, USA, #3868, clone D11, dilution 1:3000), LC3 from Novus Biologicals (Cambridge, UK, #NB600-1384, dilution 1:3000) using Tris buffer (pH 9) at 95°C for 30 minutes for antigen retrieval, p62 from MBL (IL, USA, #PM045, dilution 1:8000) using Citrate buffer (pH 6.5) at 100°C for 30 minutes for antigen retrieval. Antibody detection was done with the Bond Polymer Refine Detection kit (Leica Biosystems, DS9800) following the manufacturer's instructions.

Read out of stainings was performed by AMS and SB as established before [24], and discrepancies were discussed on a multi-header microscope to gain a final consensus.

Dot-like staining patterns for LC3 and p62 were scored as: 0 - no dots or barely visible dots in < 5% of cells, 1 - detectable dots in 5-25% of cells, 2 readily detectable dots in 25-75% of cells and 3 - dots in > 75% of cells. Stone like structures (SLS) [20] were recorded separately.

Cytoplasmic p62 staining was scored as: 0 - no or very faint staining, 1 - weak staining, 2 - moderate to strong staining. Nuclear p62 staining was recorded as present or absent.

Images were acquired using a Zeiss Axioplan 2 microscope (objective magnification 40 x and 100 x) and Axiovision software.

### Immunoblot analysis

Protein was extracted from 4 × 1 mm tumor punches using the Qproteome FFPE Tissue Kit (Qiagen, 37623) as per manufacturer's instruction. The samples were deparaffinized and rehydrated using Xylene followed by descending alcohol series. 100 μl of extraction buffer, containing β-mercaptoethanol, was added to each sample followed by ice (15 minutes) and heat treatment (100°C, 20 minutes; 80°C, 2 hours).

Samples were centrifuged and supernatant transferred to a new tube. Protein concentration was determined using the Bradford protein assay (BioRad, Cressier, Switzerland). 30 μg of total protein were denatured in 5 x self-made sample buffer, containing β-mercaptoethanol (Sigma Aldrich, M-7522, Leiden, Netherlands), at 95°C for 5 minutes. Samples were loaded and separated on a 4-20% stain-free pre-cast gel (BioRad). Total protein was visualized as loading control. Separated protein was transferred onto a polyvinylidene difluoride membrane using the Trans-Blot^®^ Turbo™ Transfer system (BioRad). Membranes were blocked in 5% bovine serum albumin (BSA)/tris-buffered saline (TBS) for 1 hour at RT. The same primary antibodies were used as for immunohistochemistry in a working concentration of 1:1000 in 5% milk/TBS with 0.1% Tween (Sigma Aldrich, P9416). Membranes were incubated with primary antibodies over night at 4°C with shaking. Subsequently, membranes were incubated with anti-rabbit IgG, HRP-linked Antibody (Cell Signaling, 7074, dilution 1:10 000 in 5% milk/TBS-T) for 3 hours at RT with shaking. Prior to visualization, membranes were incubated with Clarity Western ECL Substrate (BioRad) for 5 minutes at RT with shaking. Results were visualized using the ChemiDoc™ MP system (BioRad).

### Statistical analysis

Descriptive and comparative statistical analyses were performed using the SPSS 23 software (SPSS Inc, Chicago, IL, USA). Associations between staining patterns and clinico-pathologic parameters were evaluated using simple cross tabs (Chi^2^-test or Fisher's exact test). Survival analysis was performed using log rank test and Cox regression analysis. The significance level was set at 0.05.
